# Supplementary material for: The CLIP-170 N-terminal domain binds directly to both F-actin and microtubules in a mutually exclusive manner
Source: J Biol Chem. 2022 Mar 10;298(5):101820. doi: 10.1016/j.jbc.2022.101820 (PMC9062740; doi:10.1016/j.jbc.2022.101820)
Supplement: Supporting information [file mmc1.pdf]

Supplement to the manuscript "The N-terminal domain of the microtubule plus-end tracking protein CLIP-170 directly binds to both F-actin and microtubules in a mutually exclusive manner"

Supplement Table of Contents

Item	Description	Page
Methods	Supplementary Material and Methods	1-2
Legends	Legends for S1-S4, and the CLIP-170 alignment*	3-4
S1 Fig	CLIP-170 binds to 50 µM F-actin at physiological salt concentration (companion to Figure 1)	5
S2 Fig	F-actin and MT binding data for CLIP-170 mutants (companion to Figure 4)	6
S3 Fig	Circular dichroism (CD) data for CLIP-170 mutants	7
S4 Fig	Assessment of CLIP-170 and actin in NIH3T3 cells expressing full-length GFP-CLIP-170 (companion to Figure 7)	8
S5 Fig	Bioinformatic analysis of CLIP-170 CAP-Gly domains	9
References	Supplementary Material References	10

*The CLIP-170 alignment is provided as a separate file.

Circular Dichroism (CD)

Wild-type CLIP-170 fragments (H2, CG1, and CG2) and their mutants were dialyzed into PBS buffer (pH 7.4). The concentration of CLIP-170 fragments and their mutants was first determined by Bradford assays and confirmed by separating with 12% SDS-PAGE followed by Coomassie blue stain and intensity measurement. H2 and its mutants were diluted to 3 µM, while CG1, CG2, and their mutants were diluted to 15 µM. The spectra of buffer alone, CLIP-170 fragments, and CLIP-170 mutants were measured from 190-250 nm on a Jasco J-815 at 4°C with a 1 mm cuvette. All samples were measured three times and averaged together. The averaged spectrum data were converted to mean residue ellipticity (θ_{MRE}) by $\theta_{MRE} = \frac{M}{c \cdot l \cdot n_r} \cdot \theta_{deg}$, where M is molecular weight (g/dmol), c is concentration (g/ml), l is pathlength (cm), n_r is number of residues, and $\theta_{deg'}$ is the averaged data from CD measurement (deg).

Bioinformatics

To assess the percentage of conservation of each residue in the CLIP-170 CAP-Gly domain(s) (Figure S5(A)), we selected and aligned the sequences of CLIP-170 CAP-Gly domain(s) from a range of seven model organisms (from yeasts to humans). The selected organisms and protein sequences used were: *Homo sapiens* (taxid:9606, AAA35693.1), *Mus musculus* (taxid:10090, NP_062739.2), *Xenopus laevis* (taxid:8355, XP_018118619.1), *Danio rerio* (taxid:7955, XP_009300269.1), *Drosophila melanogaster* (taxid:7227, NP_609835.2), *Saccharomyces cerevisiae* (taxid:4932, NP_009901.1), and *Schizosaccharomyces pombe* (taxid:4896, NP_593613.1). The alignment tool was ClustalW (1).

For Figure S5(B), CAP-Gly sequences of all known CAP-Gly-containing proteins in humans were aligned. Human CLIP-170 CG1 sequence was used as the query in a BLASTp search to acquire known CAP-Gly proteins for alignment. The selected CAP-Gly sequences were: CLIP1 (NP_002947), CLIP2 (NP_003379.4), CLIP3 (NP_001186499.1), CAP350 (NP_055625.4), KIF13B (NP_056069.2), TBCB (NP_001272.2), P150 (NP_001177765.1, NP_004073.2), TBCE (NP_001072983.1), and CYLD (NP_001365672.1).

Figure S1. CLIP-170 binds to 50 μM F-actin at physiological salt concentration (companion to Figure 1). (A) Binding of 4 μM H2 to 50 μM F-actin at 75 mM or 150 mM salt concentration. The mixtures of H2, phalloidin (0.8 μM), and pre-polymerized F-actin at indicated salt concentrations were incubated for 10 min at room temperature to perform the high-speed cosedimentation assays. S indicates the supernatant; P indicates the pellet. The salt concentration corresponds to the total potassium concentration in the reaction (see Methods). 150 mM represents the physiological salt concentration (2). (B) Fraction of H2 bound to 50 μM F-actin at 75 mM or 150 mM salt concentration. Error bars represent the standard deviation (n=3). * indicates p-value < 0.05. These data indicate that H2 can bind to high concentrations of F-actin at physiological salt concentration.

Figure S2. F-actin and MT binding data for CLIP-170 mutants (companion to Figure 4). (A) Data represent the MT binding ability of each CLIP-170 mutant. High-speed co-sedimentation assays were done with 5 μM CG1, CG2, or their mutants and 10.5 μM taxol-MTs. (B) Data represent the F-actin binding ability of each CLIP-170 mutant. High-speed co-sedimentation assays were done with 2.3 μM H2 or their mutants and 10.5 μM F-actin. The concentration of proteins (CLIP-170 fragments, mutants, F-actin, and MTs) in the binding assays were chosen so that the 'fraction of CLIP-170 bound' in the controls (H2, CG1, and CG2) is approximately 0.5, which enables the most sensitive detection of affinity changes. The blue dots represent the data points. Error bars are standard deviation. The sample number of each mutant is 3 or 4, and the sample number of CLIP-170 controls is 6 (CG1), 9 (CG2) and 22 (H2). CLIP-170 controls have a higher n number because both positive and negative controls were included every time a binding assay was performed to ensure validity of the binding assay. * indicates p-value < 0.05; as described in the Results, only the subset of controls corresponding to that experiment was used to determine the indicated p-values. The open star indicates a case where the mutant appears to bind weaker than the wild-type, but the data are impacted by self-sedimentation problems, and the difference between mutant and wild-type is not significant.

Figure S3. Circular dichroism (CD) data for CLIP-170 mutants. The CD spectrum data of CLIP-170 mutants used in the binding assays in Figure 4. (A) CD data of H2 and H2 mutants used in F-actin binding assays. The signaling magnitude of H2-K70A is largely reduced, and it has severe self-pelleting so we eliminated it from the affinity study. The signaling magnitude of H2-K224A is also reduced, which might indicate some level of misfolding for this mutant, although this mutant does not have serious self-pelleting. (B) CD data of CG1 and CG1 mutants used in MT binding assays. (C) CD data of CG2 and CG2 mutants used in MT binding assays. We included two wild-type CLIP-170 fragments as positive controls and two PBS buffer-only reactions as negative controls for all three mutant sets.

Figure S4. Assessment of colocalization between GFP-CLIP-170 and F-actin in NIH3T3 cells expressing full-length CLIP-170 (companion to Figure 7). NIH3T3 cells were transfected to overexpress GFP-labeled full-length CLIP-170. Cells were fixed with methanol, and actin was probed with actin antibody. Yellow boxes outline zoom-ins that contain examples of colocalization (yellow arrows) between GFP and actin in cells expressing CLIP-170; cyan boxes outline zoom-ins that contain examples of apparent colocalization (cyan arrows) in untransfected cells, complicating interpretation of these data (similar to Figure 7). The contrast

settings of the insets are the same as those of the full images from which they are derived, enabling comparison between insets. Scale bar: 10 µm. Inset scale bar: 10 µm.

Figure S5. Bioinformatic analysis of CLIP-170 CAP-Gly domains. (A) Conservation analysis of CLIP-170 CAP-Gly domains in diverse species. CLIP-170 CG1 and CG2 sequences from a range of 7 organisms were aligned and colored by the percentage of identity using Jalview default settings. (B) Conservation analysis of CAP-Gly domains in divergent CAP-Gly-containing proteins in humans. The CAP-Gly regions in CAP-Gly containing proteins were aligned and colored by the percentage of identity using Jalview default settings. For both panels, residues mutated in Figure 4 were boxed in colors as indicated in the key, with a dot or a line to indicate single or double mutants, respectively.

CLIP170align.aln This separate file contains a machine-readable version of the CLIP-170 alignment used to generate the conservation maps in Figures 3-4. Organisms are named by the first three letters of their genus and species.

Figure S1

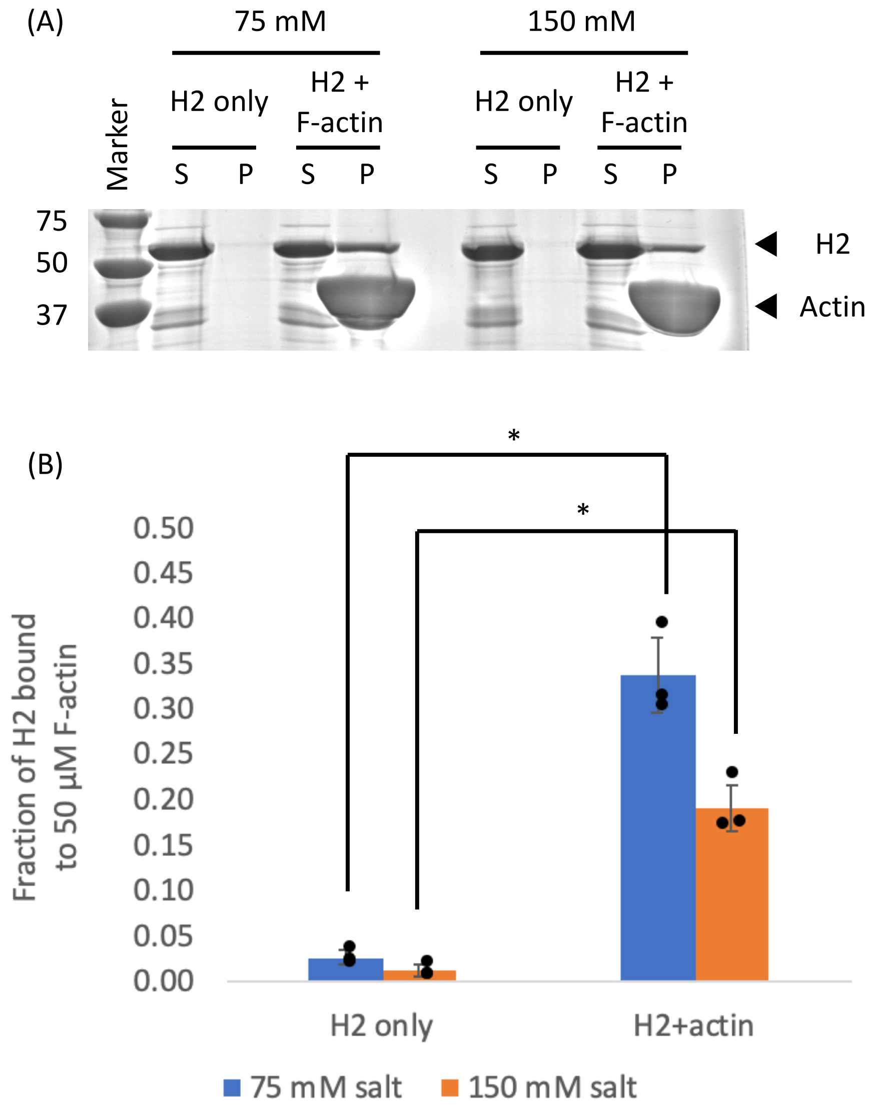

(A)
75 mM
150 mM
H2 only
H2 +
F-actin
H2 only
H2 +
F-actin
Marker
S P S P S P S P
75
50
37
H2
Actin
(B)
*
*
0.50
0.45
0.40
0.35
0.30
0.25
0.20
0.15
0.10
0.05
0.00
Fraction of H2 bound to 50 µM F-actin
H2 only
H2+actin
75 mM salt
150 mM salt

Figure S2

(A) **Binding of CG1 or CG2 fragments with mutations as indicated to MTs**

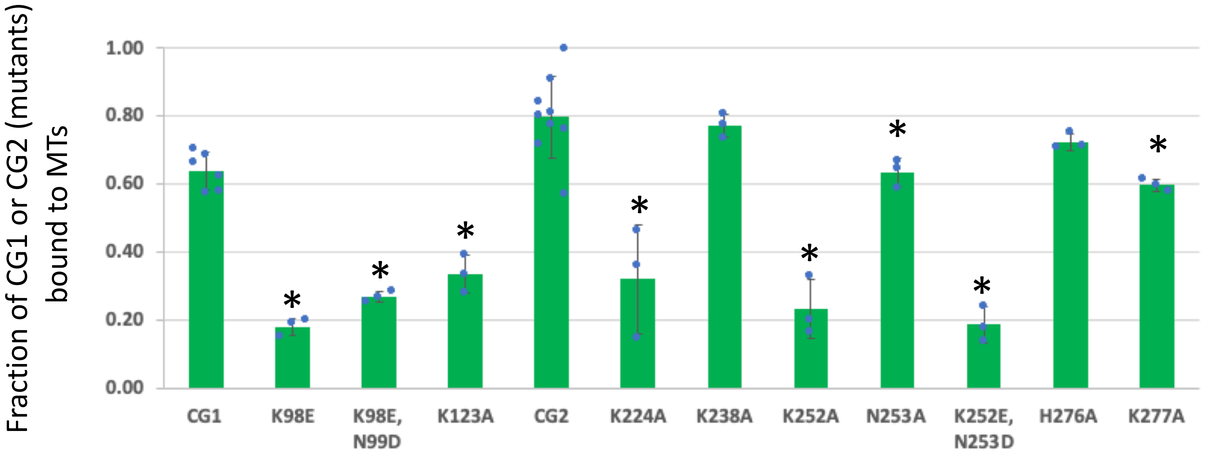

(B) **Binding of H2 fragments with mutations as indicated to F-actin**

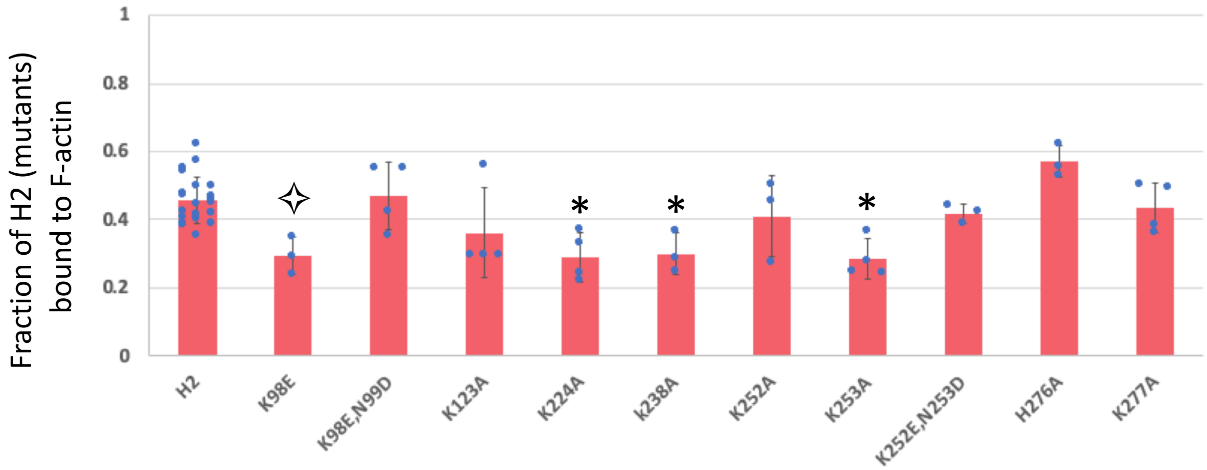

Figure S3

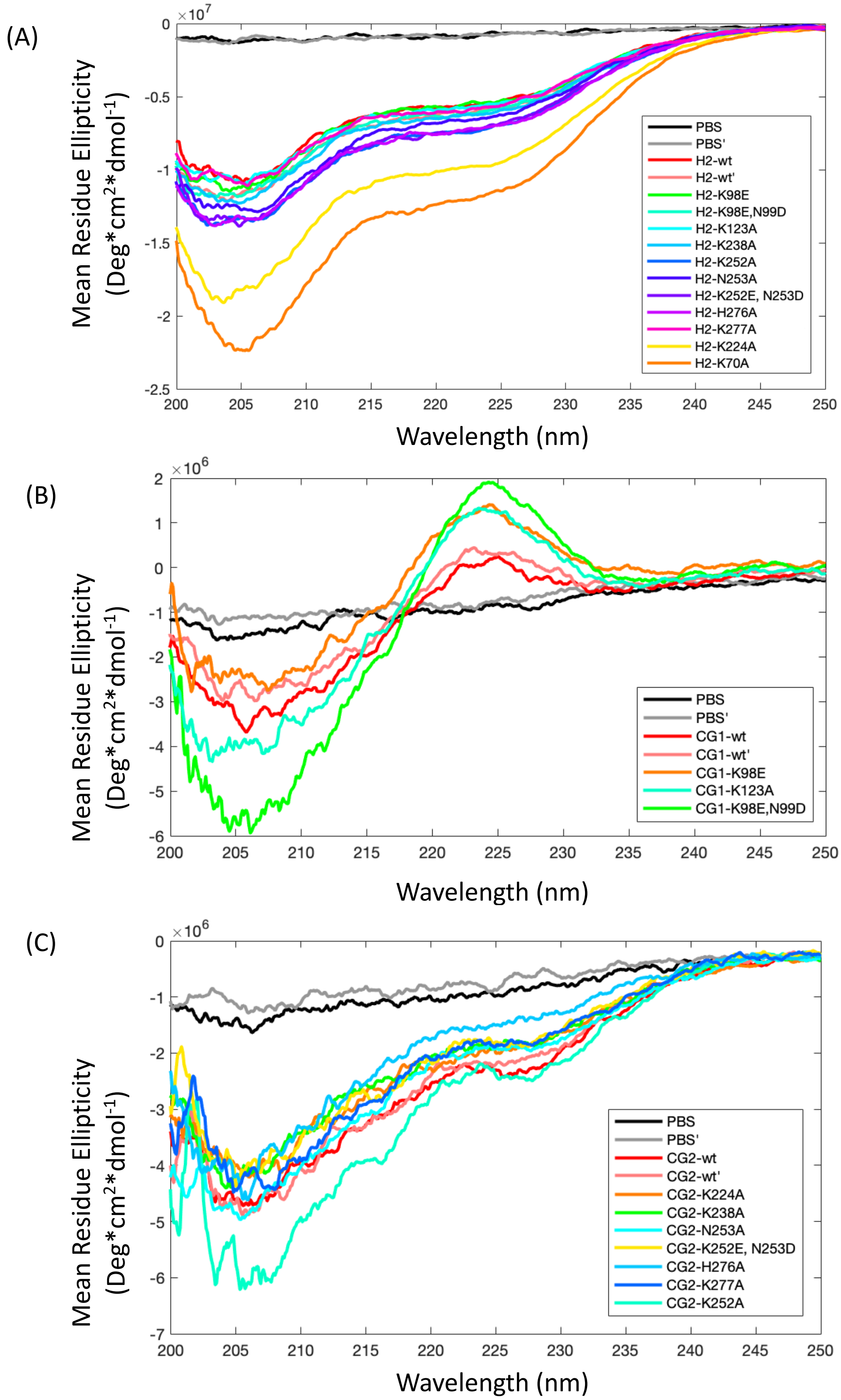
(A)
×10^7
Mean Residue Ellipticity
(Deg*cm^2*dmol^-1)
Wavelength (nm)
PBS
PBS'
H2-wt
H2-wt'
H2-K98E
H2-K98E,N99D
H2-K123A
H2-K238A
H2-K252A
H2-N253A
H2-K252E, N253D
H2-H276A
H2-K277A
H2-K224A
H2-K70A
(B)
×10^6
Mean Residue Ellipticity
(Deg*cm^2*dmol^-1)
Wavelength (nm)
PBS
PBS'
CG1-wt
CG1-wt'
CG1-K98E
CG1-K123A
CG1-K98E,N99D
(C)
×10^6
Mean Residue Ellipticity
(Deg*cm^2*dmol^-1)
Wavelength (nm)
PBS
PBS'
CG2-wt
CG2-wt'
CG2-K224A
CG2-K238A
CG2-N253A
CG2-K252E, N253D
CG2-H276A
CG2-K277A
CG2-K252A

Figure S4

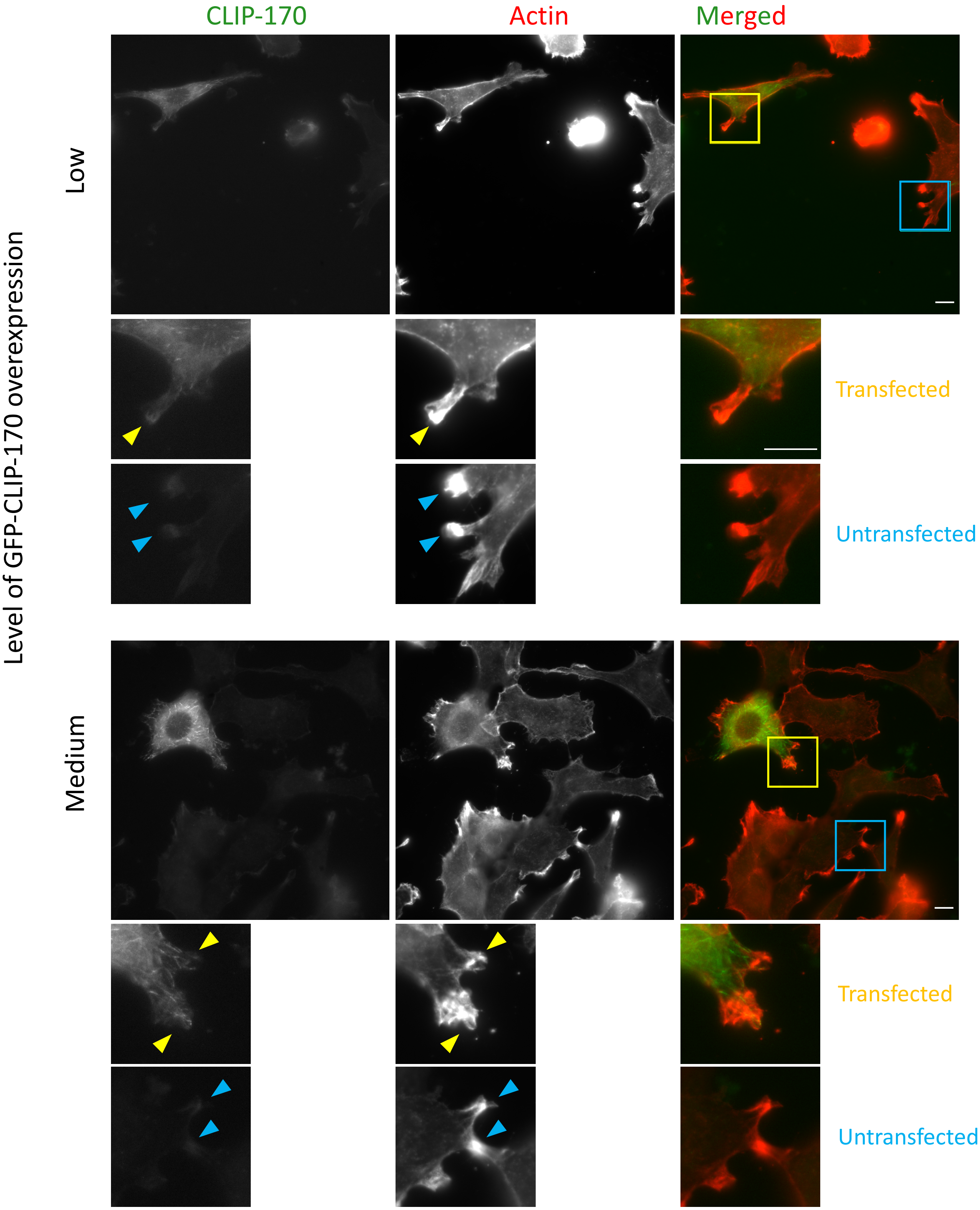
CLIP-170
Actin
Merged
Low
Medium
Level of GFP-CLIP-170 overexpression
Transfected
Untransfected
Transfected
Untransfected

Figure S5

(A) Conservation throughout species

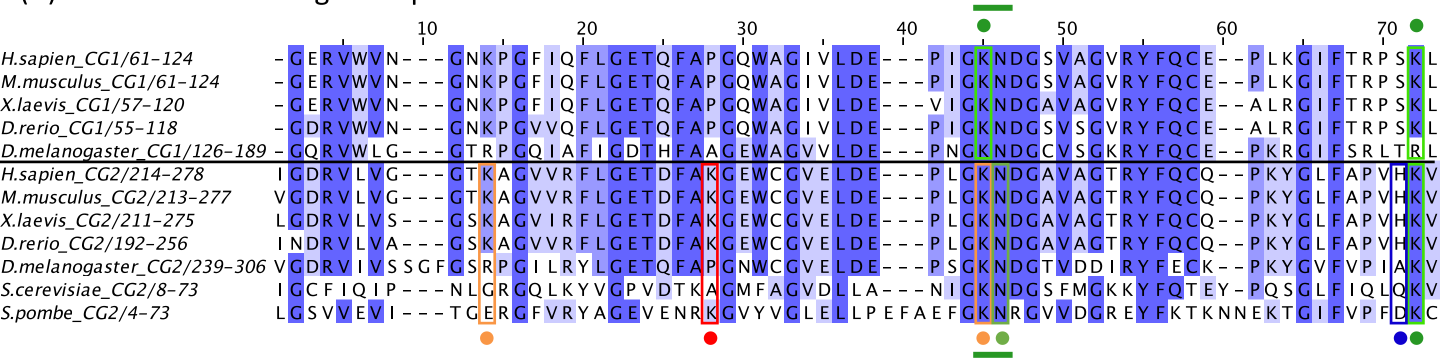

(B) Conservation throughout CAP-Gly domains

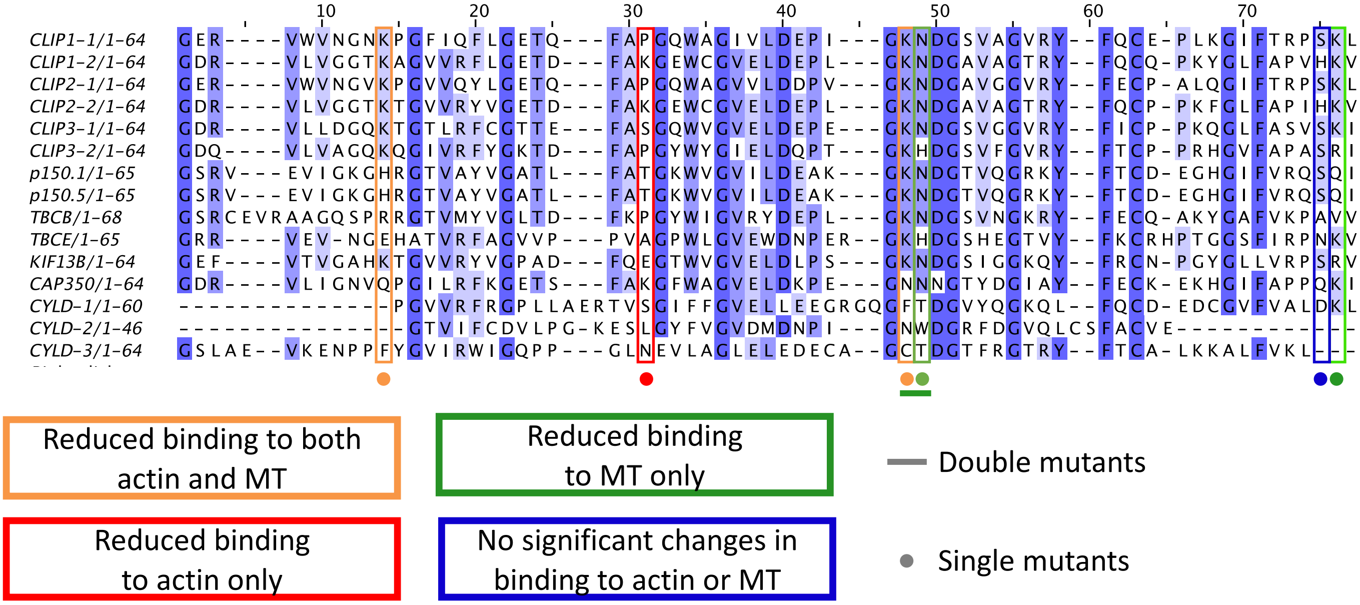

References

1. Thompson, J. D., Gibson, T. J., Plewniak, F., Jeanmougin, F., and Higgins, D. G. (1997) The CLUSTAL_X windows interface: flexible strategies for multiple sequence alignment aided by quality analysis tools. *Nucleic Acids Res* **25**, 4876-4882
2. Thier, S. O. (1986) Potassium physiology. *Am J Med* **80**, 3-7
